# Assessing the urinary concentration of nitrofurantoin and its antibacterial activity against *Escherichia coli*, *Staphylococcus pseudintermedius*, and *Enterococcus faecium* isolated from dogs with urinary tract infections

**DOI:** 10.3389/fvets.2023.1189374

**Published:** 2023-07-10

**Authors:** Chien-Che Hung, Csaba Varga, Jennifer M. Reinhart, Carol W. Maddox, Ryan N. Dilger, Lauren Forsythe, Amy K. Stevenson, Rebecca J. Franklin-Guild, Narayan C. Paul, Akhilesh Ramachandran

**Affiliations:** ^1^Veterinary Diagnostic Laboratory, College of Veterinary Medicine, University of Illinois Urbana-Champaign, Urbana, IL, United States; ^2^Department of Veterinary Clinical Medicine, College of Veterinary Medicine, University of Illinois Urbana-Champaign, Urbana, IL, United States; ^3^Department of Pathobiology, College of Veterinary Medicine, University of Illinois Urbana-Champaign, Urbana, IL, United States; ^4^Department of Animal Science, College of Agriculture, Consumer and Environmental Sciences, University of Illinois Urbana-Champaign, Urbana, IL, United States; ^5^Animal Health Diagnostic Center, College of Veterinary Medicine, Cornell University, Ithaca, NY, United States; ^6^Texas A&M Veterinary Medical Diagnostic Laboratory, College Station, TX, United States; ^7^Oklahoma Animal Disease Diagnostic Laboratory, College of Veterinary Medicine, Oklahoma State University, Stillwater, OK, United States

**Keywords:** nitrofurantoin, dog, UTI, breakpoint, *Escherichia coli*, *Staphylococcus*, *Enterococcus*, macrocrystalline

## Abstract

Nitrofurantoin, a broad-spectrum nitrofuran class antibiotic, is applied as a first-line antibiotic in treating human urinary tract infections (UTIs) due to its great efficacy and high achievable concentration. The interest in using this antibiotic in companion animals has increased due to the growing demand for effective antibiotics to treat UTIs caused by multidrug-resistant bacteria. Currently, the susceptibility interpretations for nitrofurantoin are based on the breakpoints set for humans, while the canine-specific breakpoints are still unavailable. In this study, we assessed the concentration of nitrofurantoin reaching the dog’s urine using the recommended oral dosing regimen. In addition, we examined the efficacy of this breakpoint concentration against the common canine UTI pathogens, *Escherichia coli, Staphylococcus pseudintermedius*, and *Enterococcus faecium*. Eight experimental beagle dogs were treated with ~5 mg/kg of nitrofurantoin macrocrystal PO 8qh for 7 days. The urine samples were collected via cystocentesis at 2, 4, and 6 h after administration on day 2 and day 7 and used to quantify nitrofurantoin concentrations by ultra-high performance liquid chromatography. The results showed that 26.13–315.87 μg/mL nitrofurantoin was detected in the dogs’ urine with a mean and median concentration of 104.82 and 92.75 μg/mL, respectively. Additionally, individual dogs presented with urinary nitrofurantoin concentrations greater than 64 μg/mL for at least 50% of the dosing intervals. This concentration efficiently killed *E. coli*, and *S. pseudintermedius*, but not *E. faecium* strains carrying an MIC_90_ value equal to 16, 16, and 128 μg/mL, respectively. Taken together, these results suggest that the value of 64 μg/mL may be set as a breakpoint against UTI pathogens, and nitrofurantoin could be an effective therapeutic drug against *E. coli* and *S. pseudintermedius* for canine UTIs.

## Introduction

Lower urinary tract infections (UTIs) are one of the most common diseases in companion animals (1, 2). The emergence of multidrug-resistant (MDR) urinary bacterial pathogens has negatively affected the treatment efficacy of canine UTIs (3–5). A recent research study in Illinois assessed the prevalence of major bacterial pathogens and their antibiotic resistance patterns in canine urine samples submitted to a veterinary diagnostic laboratory, identifying *Escherichia coli* as the most common among Gram-negative bacteria, and *Staphylococcus pseudintermedius* and *Enterococcus* sp. among Gram-positive bacteria (5). These urinary pathogens revealed a high frequency of resistance to first-line antibiotics used to treat UTIs, such as ampicillin, amoxicillin-clavulanic acid, and trimethoprim-sulfamethoxazole (5). Additionally, the transmission of MDR UTI pathogens from companion animals to humans also raises zoonotic concerns (6). Thus, identifying an effective antibiotic to treat resistant UTI pathogens in dogs is desperately needed.

Nitrofurantoin, a synthetic nitrofuran, has been used as a first-line antibiotic to treat human UTIs due to its broad-spectrum bactericidal activity, effectiveness against several Gram-positive and Gram-negative bacteria, and its ability to achieve a high concentration in urine (7, 8). However, the other nitrofuran class antibiotic, nitrofurazone was only applied for topical treatment in dogs, cats, and horses, and banned for systemic treatment due to the carcinogenicity concern (9–11). Nitrofurantoin’s popularity has increased in companion animal medicine because of the increasing demand for effective antibiotics to treat UTIs caused by MDR bacteria, such as methicillin-resistant *S. pseudintermedius* (MRSP) and extended-spectrum beta-lactamase (ESBL) producing *E. coli* (7). Additionally, due to its low-cost and convenient oral administration, nitrofurantoin becomes a useful alternative for extra-label treatment of these specific MDR urinary pathogens.

In clinical practice to treat canine UTIs, nitrofurantoin is recommended orally at a 4.4–5 mg/kg dose, three times daily (12, 13). This antibiotic has a bioavailability of 38–120% in dogs via oral administration with a short terminal half-life of 19–87 min in the serum (14). The drug is primarily eliminated into the urine via glomerular filtration and renal tubular secretion in its native form (15–17). Some nitrofurantoin can be reabsorbed at the renal tubular level if the urine is at a lower pH value, thereby reducing its concentration reaching the urinary bladder (17). A small portion of the drug undergoes enterohepatic recirculation and is eventually eliminated through urine or feces (14, 18). The absorption and duration in the plasma and urine of this drug has been shown to vary due to different formulations, crystal size, and duration in the gastrointestinal tract (19). Macrocrystalline nitrofurantoin is believed to provide steady levels of concentration in the urine and exerts fewer gastrointestinal side effects due to slow release in the gastrointestinal tract (19). Although a prokinetic study of nitrofurantoin microcrystals with the recommended dosing regimen has been reported in dogs (20), the concentration of nitrofurantoin and the persistence of the drug in urine by treating the dogs with the macrocrystalline form is still unclear. Knowing these parameters is crucial to assessing its potential efficacy against bacterial pathogens causing canine UTIs. In addition, the interpretation of susceptibility to nitrofurantoin for bacterial pathogens in canine UTIs currently uses breakpoints set for humans with 32, 64, and 128 μg/mL as sensitive, intermediate, and resistant, respectively (21, 22). However, canine-specific breakpoints are still unavailable. Additionally, evaluating the validity of applying human breakpoints to dogs needs to be established to correctly determine nitrofurantoin susceptibility (sensitive, intermediate, or resistant) for bacteria isolated from clinical canine urine samples.

To assess the potential efficacy of nitrofurantoin in treating canine UTIs, our study objectives are to (1) measure the urinary concentrations of nitrofurantoin in dogs that can be achieved through a 7-day oral administration of macrocrystalline nitrofurantoin and establish a clinically relevant breakpoint; (2) define the MIC_50_ and MIC_90_ values of common UTI bacteria: *E. coli*, *S. pseudintermedius,* and *Enterococcus faecium*; (3) determine the *in vitro* antibacterial activity of nitrofurantoin against these pathogens in canine urine; (4) assess the side effects of treatment.

## Materials and methods

### Determining achievable nitrofurantoin concentrations in dog urine and establishing a minimum inhibitory concentration breakpoint

**Figure 1:**
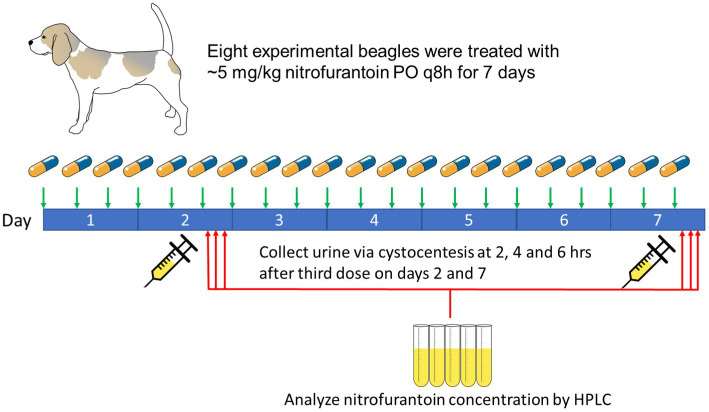
Experimental design to evaluate the concentration of nitrofurantoin in dogs’ urine.

To establish achievable nitrofurantoin concentration in the urine of normal dogs, eight adult purpose-bred female spayed beagle dogs with a weight range of 8.75–10.9 kg and a median weight of 9.95 kg were included in the study (Figure 1). All dogs had no UTI nor any drug use history 30 days before the experiment. The serum biochemistry profile and urinalysis were examined 3 days before initiating and after finishing the experiment for all study dogs to verify normal hepatic and renal parameters. Each dog received a 50 mg nitrofurantoin macrocrystal capsule (Zydus Pharmaceuticals, NJ, United States) embedded in a 30 g canned food meatball (achieving ~5 mg/kg of nitrofurantoin) by mouth and 140 g kibble as a meal every 8 h for 7 days. Urine samples were collected via ultrasound-guided cystocentesis at 2, 4, and 6 h after the third administration on day 2 and day 7. After collection, all samples were protected against light and stored at −80°C to avoid degradation. The side effects caused by nitrofurantoin, including vomiting, diarrhea, and loss of appetite, were also monitored throughout the entire experimental period. This animal experiment was approved by the Institutional Animal Care and Use Committee at the University of Illinois at Urbana-Champaign with protocol no. 20242.

The minimum inhibitory concentration (MIC) breakpoints have been applied as a universal standard for susceptibility interpretation. To determine the potential MIC breakpoint for canine urine, nitrofurantoin concentrations in each dog were further analyzed. Traditionally, drug concentrations for time-dependent drugs should exceed the MIC of the organisms for at least 50% of the dosing interval (23). Thus, these criteria were used to establish a MIC breakpoint in urine for orally administered nitrofurantoin in dogs based on the observed concentrations.

### Measuring the concentration of nitrofurantoin in the dog’s urine by high-performance liquid chromatography

The concentration of native-form nitrofurantoin in each of the urine samples was measured by high-performance liquid chromatography (HPLC) (24, 25).

To generate a standard curve, drug-free sterile (confirmed by bacterial culture) urine from one of the experimental beagle dogs was spiked with nitrofurantoin to create solutions containing final concentrations of the drug ranging from 0.05–300 μg/mL. A final concentration of 20 μg/mL of furazolidone was also included in each standard and sample as an internal standard. The linearity of the detection range (0.05–300 μg/mL) of nitrofurantoin was adequate (R^2^ = 0.9993). This validated technique met all procedural requirements in terms of specificity, sensitivity, and repeatability, thereby allowing precise and accurate quantification of nitrofurantoin in canine urine.

### Determining MIC_50_ and MIC_90_ values of nitrofurantoin for *Escherichia coli*, *Staphylococcus pseudintermedius*, and *Enterococcus faecium* isolates

A total of 108 *E. coli*, 108 *S. pseudintermedius*, and 106 *E. faecium* clinical isolates obtained from dog urine samples submitted to veterinary diagnostic laboratories across the United States were included in the study. Each bacterial strain was identified by matrix-assisted laser desorption/ionization-time of flight mass spectrometry with a score > 2.0, indicating the accuracy of results to species level.

Each isolate was tested for MIC of nitrofurantoin by using the broth dilution method with testing concentrations ranging from 1 to 512 μg/mL in two-fold dilutions following the Clinical and Laboratory Standards Institute guidelines (21, 22). Further, the MIC_50_ and MIC_90_ cutoff values of nitrofurantoin were calculated for each of the three bacterial species. The MIC_50_ signified the concentration that inhibited the growth of 50% of isolates, while the MIC_90_ signified the concentration at which 90% of isolates’ growth was inhibited.

### Evaluating the *in vitro* bactericidal activity of nitrofurantoin

Five isolates of *E. coli*, *S. pseudintermedius* or *E. faecium* carrying a MIC value equal to the MIC_90_ were added to the 0.22 μM filter sterilized dog urine (pH = 5.2) collected from a 4-year-old healthy female spayed German Shorthaired Pointer (the dog had no history of UTI or urogenital diseases and was not under any medication 2 weeks before collecting the urine) containing 64 μg/mL of nitrofurantoin (the breakpoint established in the previous experiments). After 0, 4, 8, 16, and 24 h of incubation, the bacterial suspension was diluted by 10-fold serial dilutions and plated on LB agar in triplicate to determine the surviving bacterial numbers.

### Statistical analysis

Statistical analyses were conducted by using the STATA/IC software (Version 14.2, Stata Corporation, College Station, TX). For each dog, median and mean concentrations of nitrofurantoin were calculated.

A linear regression model was constructed that accounted for the repeated measures of dogs to compare the concentration of nitrofurantoin on day 2 and day 7. Linear predictions for nitrofurantoin concentration and their 95% confidence intervals on day 2 and day 7 were calculated and illustrated in a graph.

## Results

### Assessing the urinary concentration of nitrofurantoin and establishing a MIC breakpoint

**Figure 2:**
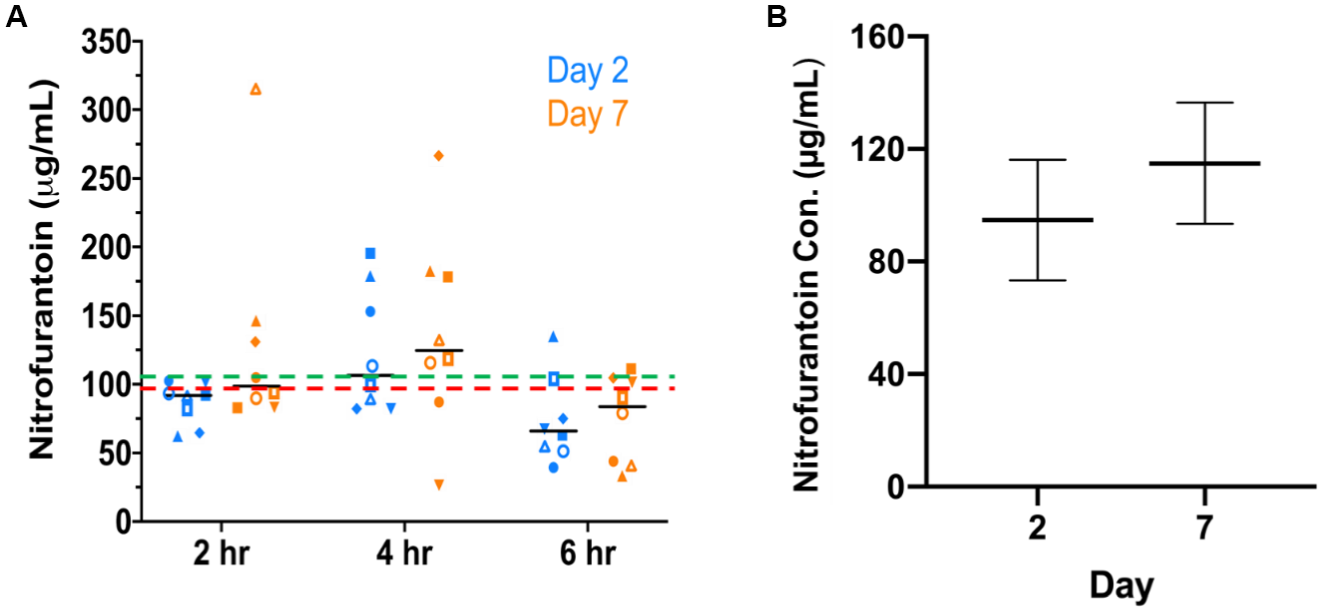
Nitrofurantoin concentration detected in dogs’ urine after oral administration. **(A)** The urinary concentration of nitrofurantoin was detected by HPLC at 2, 4, and 6 h after the third dose on day 2 and day 7. The red dash line (---) and the green dash line (---) illustrate the median (92.75 μg/mL) and mean (104.82 μg/mL) concentration in eight dogs’ urine, respectively. The black bars show the median concentration at each time point. **(B)** Adjusted predictions of concentration of nitrofurantoin with 95% CIs on days 2 and 7 in eight experimental dogs after oral administration of the drug.

As shown in Figure 2A, nitrofurantoin concentrations ranging from 26.13–315.87 μg/mL were detected in dog urine with a mean value of 104.82 μg/mL and a median value of 92.75 μg/mL. The linear predictions of nitrofurantoin concentration (Figure 2B), accounting for repeated measures of dogs, were 94.73 μg/mL (95% CI: 73.24–116.23) on day 2 and 114.90 μg/mL (95% CI, 93.40–136.40) on day 7. Although slightly higher concentrations of nitrofurantoin were detected in the dog urine on day 7, no significant differences were seen between collection times (2 vs. 4 vs. 6 h) or days (day 2 vs. 7). Throughout the entire treatment course, no obvious side effects such as vomiting or diarrhea, were observed in the individual dogs. Only one dog each showed a slight loss of appetite on day 4 (No. 4 dog on one meal) and day 5 (No. 6 dog on two meals). However, this symptom was completely resolved the next day.

**Figure 3:**
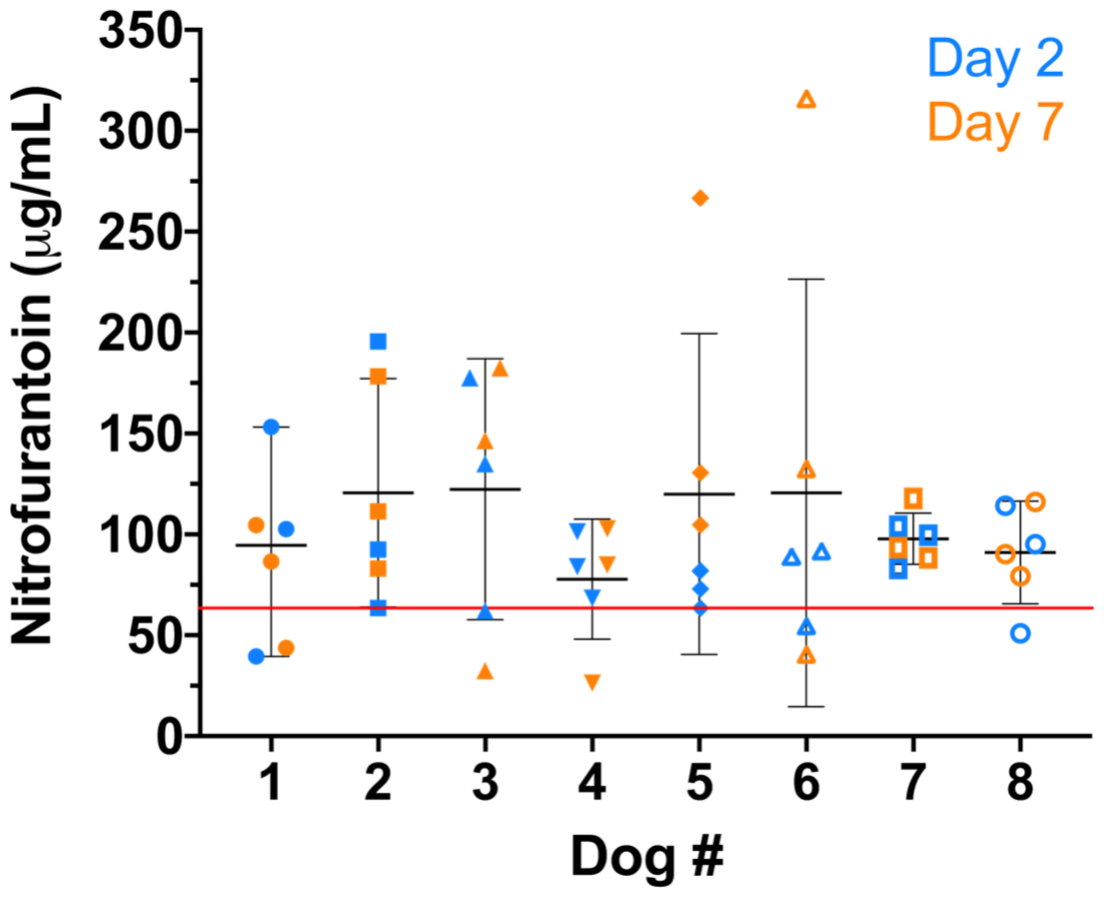
Nitrofurantoin concentration of each dog (*n* = 8). The blue and orange symbols represent the nitrofurantoin concentrations detected on day 2 and day 7, respectively. The black bar shows the median concentration of each dog. The red line illustrates the breakpoint 64 μg/mL established for canine urine samples.

Additionally, we found that nitrofurantoin concentrations equal to or greater than 64 μg/mL were achieved in at least 50% of the dosing intervals in each dog (Figure 3). These results suggest that 64 μg/mL may be set as the baseline effective concentration and the MIC breakpoint for nitrofurantoin.

### Determining MIC distribution and the MIC_50_ and MIC_90_ cutoff values for nitrofurantoin

**Figure 4:**
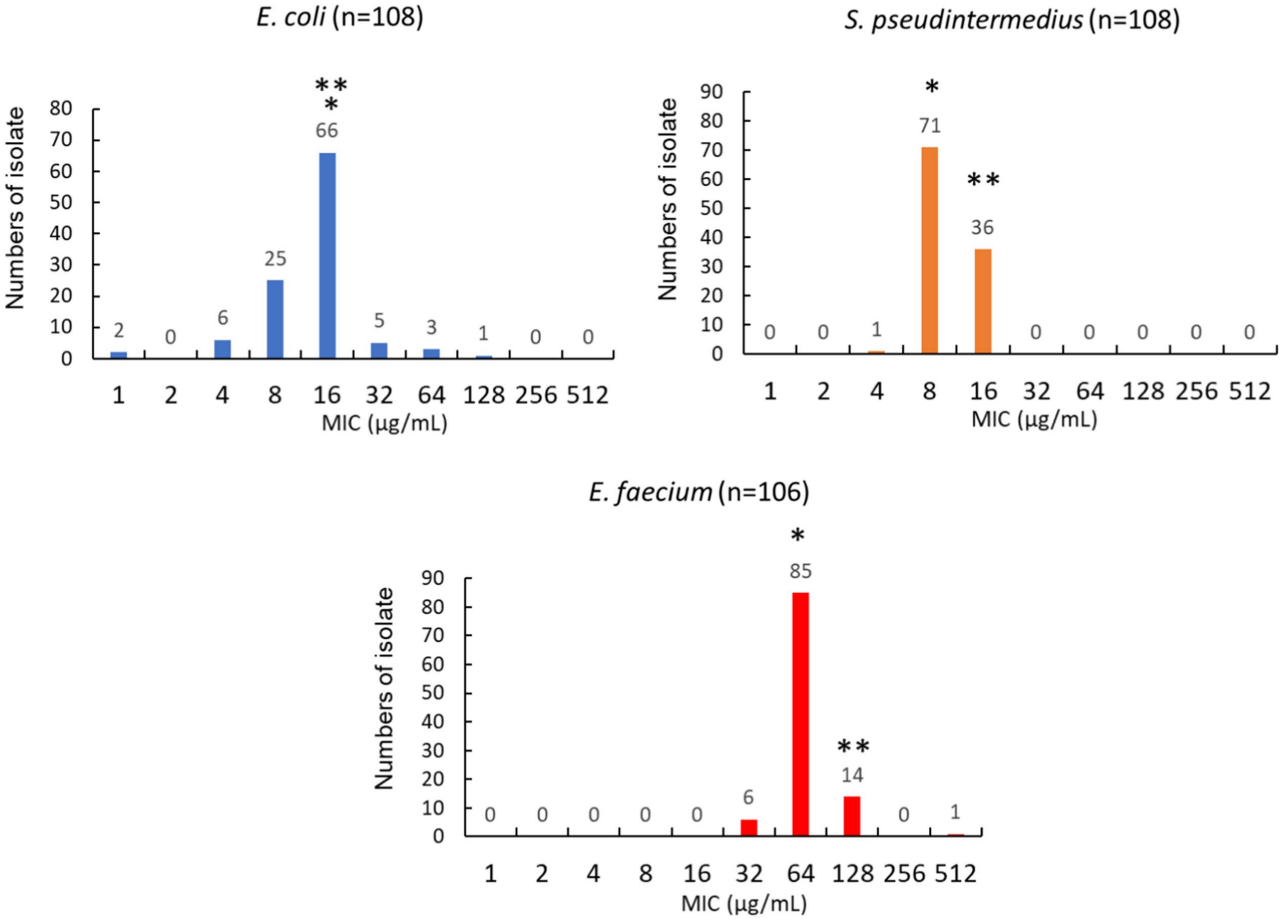
Distribution of MIC values for *Escherichia coli*, *Staphylococcus pseudintermedius*, and *Enterococcus faecium* isolated from dogs’ urine. The * represents the value of MIC_50_. The ** represents the value of MIC_90_.

To evaluate whether nitrofurantoin could be an effective antibiotic against common UTI pathogens in dogs, we also examined the MIC distribution of nitrofurantoin in at least 100 clinical isolates of each of the following: *E. coli, S. pseudintermedius,* and *E. faecium*. The MICs of nitrofurantoin ranged between 1 and 128 μg/mL for *Escherichia coli*, between 4 and 16 μg/mL for *S. pseudintermedius,* and between 32 and 512 μg/mL for *E. faecium* (Figure 4). The MIC_50_ value of *E. coli*, *S. pseudintermedius*, and *E. faecium* were 16, 8, and 64 μg/mL, respectively. Both *E. coli* and *S. pseudintermedius* had a MIC_90_ of 16 μg/mL. In addition, 96% (104/108) of *E. coli* and 100% (108/108) of *S. pseudintermedius* carried a MIC less than the proposed breakpoint of 64 μg/mL for nitrofurantoin in dog urine. In contrast, *E. faecium* had a MIC_90_ of 128 μg/mL, and only 6% (6/106) of the isolates possessed a MIC less than the proposed breakpoint.

### Evaluating the *in vitro* bactericidal activity of nitrofurantoin

**Figure 5:**
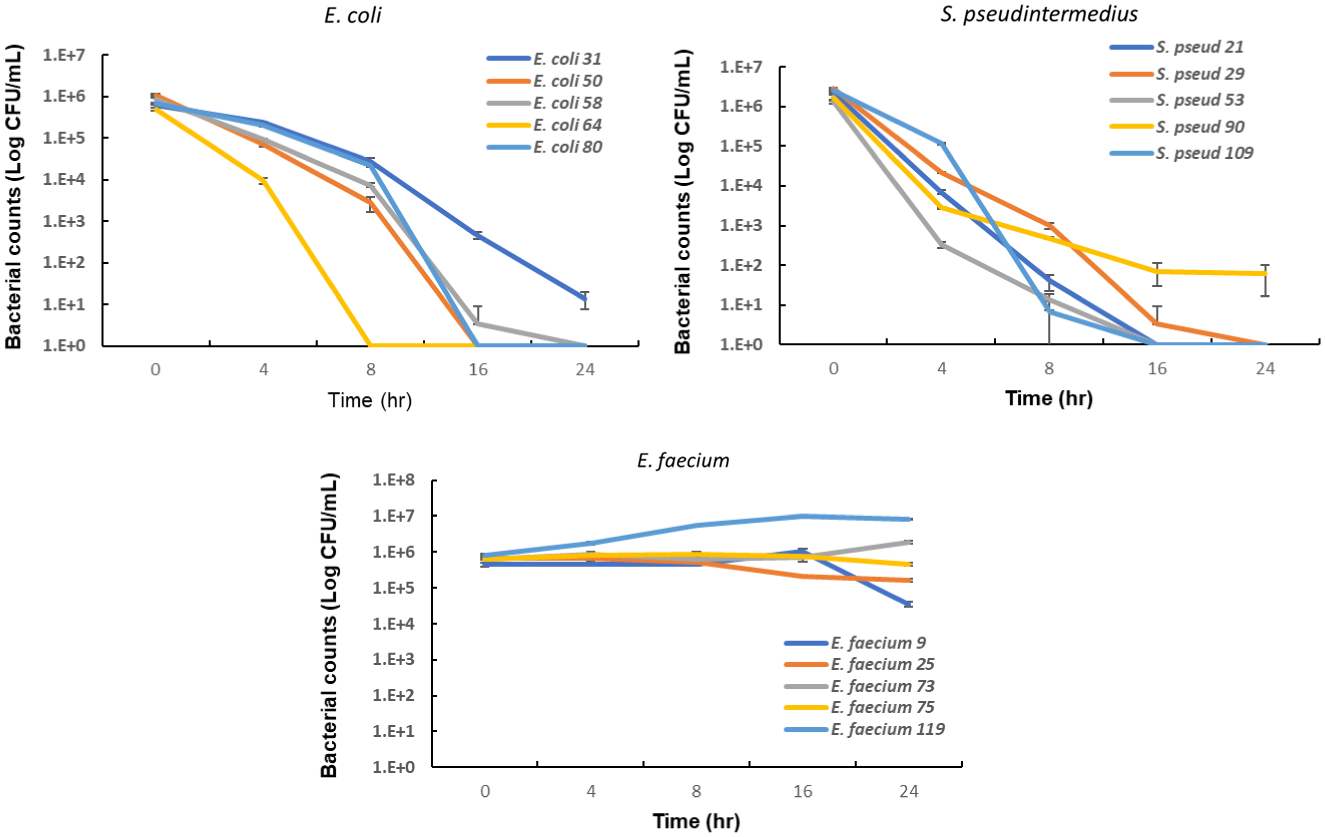
*In vitro* bactericidal activity of nitrofurantoin against common urinary bacterial pathogens. The error bars illustrate the standard deviation of bacterial counts in triplicate.

To evaluate the potential clinical efficacy of nitrofurantoin, we also performed an *in vitro* bacterial killing assay to mimic *in vivo* conditions. We used urine containing 64 μg/mL of nitrofurantoin and added the *E. coli*, *S. pseudintermedius*, or *E. faecium* strains carrying the MIC value equal to the MIC_90_. As shown in Figure 5, after 24 h of incubation, a significant 4–6 log_10_ reduction in bacterial numbers for *E. coli* and *S. pseudintermedius* was observed. For *E. faecium*, in contrast, the bacterial counts remained high in the urine (Figure 5), indicating little to no effect of this antibiotic on this organism.

## Discussion

This study assessed the urinary concentration of nitrofurantoin in experimental beagle dogs after administration of a ~5 mg/kg dose of nitrofurantoin orally, every 8 h for 7 days. Our results indicate that nitrofurantoin urinary concentrations exceed 64 μg/mL for greater than 50% of the dosing interval, suggesting that this concentration may be established as the breakpoint for treating susceptible urinary pathogens. The MIC_90_ breakpoints of nitrofurantoin for canine urinary isolates of *E. coli* and *S. pseudintermedius* were well below this proposed breakpoint. This concentration (64 μg/mL of nitrofurantoin) also efficiently killed *E. coli* and *S. pseudintermedius* strains carrying MICs equal to the species’ MIC_90_ in an *in vitro* cystitis simulation. Taken together, these results suggest that nitrofurantoin could be an effective antibiotic for these two common pathogens causing UTIs in dogs.

Our study showed a higher concentration of nitrofurantoin can be reached in canine urine compared to the results reported by Ekstrand et al. (20). Although both studies were performed using the recommended dosing regimen, the difference could be a result of using different crystal sizes in each study; macrocrystals vs. microcrystals, as well as using different pharmaceutical manufacturers. The macrocrystalline form is recommended in human medicine since it maintains nitrofurantoin concentration better and has fewer gastrointestinal side effects (19), which had been the major concern for this antibiotic in companion animals. There are few studies on the safety of nitrofurantoin used to treat bacterial cystitis in dogs. Leuin et al. recently reported in a case study where 14 dogs had successful treatment categorized as a bacteriologic cure documented in 9 of 12 dogs and included resolution of methicillin-resistant *S. pseudintermedius* (MRSP; *n* = 3), extended-spectrum β-lactamase-producing *E. coli* (2), MDR *E. coli* (1), *Enterococcus* spp. (1), MDR *E. coli* with MRSP (1), and *E. coli* with *Enterococcus* spp. (1) (26). Nitrofurantoin-associated side effects occurred in one dog. This dog experienced tolerable gastrointestinal side effects (diarrhea) and continued nitrofurantoin (3.6 mg/kg PO q8h × 7 days) to achieve treatment success with a negative urine culture. The diarrhea was resolved with the discontinuation of nitrofurantoin. Ekstrand et al. observed a lack of adverse effects among 8 healthy beagles even following the administration of nitrofurantoin microcrystals at the recommended dose for 5 days (20). In our study, we did not observe any serious gastrointestinal disturbances, such as vomiting or diarrhea throughout the 7-day treatment course. Only two dogs showed a slight loss of appetite on 1 day, and this symptom was resolved within 24 h. Therefore, these results, taken together, suggest that administering nitrofurantoin with the recommended dosing regimen is a safe and effective approach for treating sporadic bacterial cystitis in dogs.

Nitrofurantoin is commonly utilized as prophylaxis treatment for recurrent UTIs in humans (27). However, we recommend that it should be given to dogs only as needed with a singular ≤ 14-day therapeutic course. This caveat is due to the lack of a comprehensive safety study on long-term administration in companion animals, particularly considering its gastrointestinal side effects and the carcinogenic potential shown in other nitrofurans. Additionally, the prophylactic application in canine UTIs could select for nitrofurantoin resistant organisms. This could further increase the risk for the exchange of resistant UTI pathogens between humans and animals.

Our results showed that nitrofurantoin did not inhibit the growth of *E. faecium* isolates in our study and could not kill the organism after a prolonged incubation period of 24 h (Figure 5). The MIC_90_ of this drug for our isolates of this bacterial species was higher than the concentration used. These results indicate that nitrofurantoin should not be used empirically against *E. faecium* and only be used for treatment if the isolates are susceptible to nitrofurantoin. In contrast to *E. faecium*, our previous study showed no resistance in the other most common Enterococcal UTI pathogen, *E. faecalis* (5). Thus, knowing the species of the *Enterococcus* isolate is important, as the species-associated resistance pattern may lead to distinctive nitrofurantoin treatment outcomes. In addition, studies have shown that some UTI pathogens can be resistant to this drug. *Proteus*, *Pseudomonas*, *Serratia*, and *Morganella* are known to be intrinsically resistant to nitrofurantoin (28). A high prevalence of resistance to nitrofurantoin has also been reported in species of *Klebsiella*, *Citrobacter,* and *Enterobacter* (28–30). Based on these reports, identifying the species of causative pathogens and their antimicrobial susceptibility will be critical to attaining effective therapy. Another factor that can affect the therapeutic effect of nitrofurantoin is the pH value of urine. Low pH is known to facilitate its efficacy against bacterial uropathogens (31, 32). Thus, a urine acidifier might be applied to enhance its antimicrobial activity. However, as aforementioned, the low pH can simultaneously decrease the concentration of nitrofurantoin available to the lower urinary tract (17). Avoiding over adjustment could be important to attain the expected clinical efficacy of this drug.

We showed that a small percentage of *E. coli* isolates showed resistance to nitrofurantoin (Figure 4). To exert the antimicrobial ability of nitrofurantoin, it must first be converted to a toxic intermediate inside the bacterial cytoplasm (33, 34). In *E. coli*, this activation requires nitroreductase, NfsA, and NfsB (33). Nonsense mutation or coding sequence shifting on these two nitroreductase genes or on *ribE*, which is involved in the biosynthesis of the co-factor of NfsA and NfsB, could confer resistance to nitrofurantoin (33, 34). The plasmid-encoded efflux pump *OqxAB* also contributes resistance by reducing the toxic intermediate inside the bacterial cytoplasm (35). These resistance mechanisms were also suggested to apply to other species within *Enterobacteriaceae* (30). Future investigations on the mutational landscape and the plasmid-mediated efflux pumps in veterinary clinical isolates will provide valuable information for monitoring the transmission of nitrofurantoin resistance in animals and humans and preventing new resistance emergence, thereby enhancing the theme of One Health.

## Conclusion

Nitrofurantoin is a safe and effective antibiotic to treat *E. coli* and *S. pseudintermedius* induced UTIs in dogs. Administering macrocrystalline nitrofurantoin with a dosing regimen of ~5 μg/mL PO q8h, this antibiotic presented and maintained good concentrations in the dogs’ urine during a 7-day treatment course. A 64 μg/mL concentration of nitrofurantoin was detected in individual dogs’ urine for at least 50% of the dosing intervals. This concentration is considered to be the threshold with clinical efficacy in dog UTI cases, and can further be applied as the MIC breakpoint for the interpretation of nitrofurantoin susceptibility in canine urine specimens.

## Data availability statement

The original contributions presented in the study are included in the article/supplementary material, further inquiries can be directed to the corresponding author.

## Ethics statement

The animal study was reviewed and approved by Institutional Animal Care and Use Committee at the University of Illinois at Urbana-Champaign with protocol no. 20242.

## Author contributions

C-CH, JR, CV, CM, and LF: conceptualization. C-CH, JR, CV, CM, and AS: investigation. C-CH, CM, and RD: methodology. C-CH and CV: formal analysis and visualization. C-CH, CV, CM, and JR: writing-original draft. C-CH, JR, CV, CM, LF, RD, RF-G, NP, and AS: review and editing. C-CH, RF-G, NP, and AR: resources. C-CH: supervision and project administration. All authors contributed to the article and approved the submitted version.
